# Short-term effects of nitrogen deposition on nitrogen spatial and temporal distributions in a *Calamagrostis angustifolia* wetland of the Sanjiang Plain

**DOI:** 10.1371/journal.pone.0232767

**Published:** 2020-05-21

**Authors:** Xiaoling Fu, Hongwei Ni, Yingnan Liu, Jifeng Wang, Jianbo Wang, Fang Ma

**Affiliations:** 1 Harbin Institute of Technology, Harbin, China; 2 Heilongjiang Academy of Sciences Institute of Natural Resources and Ecology, Harbin, China; 3 Heilongjiang Academy of Foresty, Harbin, China; Shandong University, CHINA

## Abstract

Nitrogen (N) availability is an important factor regulating the feedback mechanisms of global change. This research uses a small *Calamagrostis angustifolia* wetland i = on the Sanjiang Plain of Northeast China as the research object and ^15^N tracer technology to study the effects of different nitrogen deposition levels (0 gN/m^2^, 4 gN/m^2^, and 8 gN/m^2^) through in situ controlled field experiments. Temporal and spatial distribution patterns of nitrogen in plants and soils and their short-term effects on nitrous oxide emissions fluxes were studied. The results showed that 1) the nitrogen content in the stems, leaves and roots of *C*. *angustifolia* decreased slowly with the growing season. Nitrogen application significantly increased the absorption of tracer nitrogen in the aboveground and underground plant parts (P<0.01), and the more nitrogen applied, the larger the absorption amount was (P<0.01). The absorbed amount accounted for 52%-86% of the total tracer nitrogen. 2) The tracer nitrogen in the soil did not show a significant change; the more nitrogen that was applied, the more nitrogen that was retained in the soil, and the tracer nitrogen adsorbed by the soil was mainly ammonium nitrogen. 3) The variation in the ^15^N-labeled nitric oxide emissions flux under different nitrogen treatments was consistent; nitrogen application increased the ^15^N-labeled nitric oxide emissions flux, but the difference between the low-nitrogen and high-nitrogen treatments was not significant (P>0.05).

## 1. Introduction

With the increase in chemical fertilizer consumption and the continuous utilization of fossil fuels, excessive nitrogen has been deposited in the plant ecosystem through the atmosphere and then stored in the soil through leaching, as part of the nitrogen cycle of the ecosystem [1,2]. Nitrogen is absorbed from the air through photosynthesis in tissues, causing nitrogen accumulation in various organs of plants, such as stems and leaves [3,4] Most scholars consider soil nitrogen storage, absorption, and loss and soil microbial nitrification and denitrification to determine the impact of the nitrogen cycle on ecosystems. The increase in emissions and sedimentation of reactive nitrogen has already had a major impact on the processes and characteristics of various ecosystems [5,6,7,8,9].Wetlands are ecosystems with strong land and water interactions and mainly include natural or artificial swamps, peatlands and waters. As important ecosystems of the land-sea transition zone, they play an important role in conserving water resources, decreasing water-level peaks and floods, regulating climate, purifying pollutants, maintaining soil and water, storing carbon pools, and providing habitats for species given their special composition and structure. Wetlands provide environmental protection and other types of protection. As a unique ecosystem, wetlands play an important role in the global change process. The Sanjiang Plain contains the most intact and well-maintained original wetland in China. This wetland has high biodiversity and is also an important representative of wetland ecosystems and a wetland of international significance [10]. *C*.*angustifolia* is a dominant plant in the typical meadows and swampy meadows on the Sanjiang Plain. This plant is typically a dominant species, subdominant species or important associated species in marsh vegetation [11,12,13]. Therefore, this study used the typical wetland of the Sanjiang Plain, a *C*. *angustifolia* wetland, as the research object and used the ^15^N tracer technique to study the spatial and temporal distribution patterns of nitrogen in plant-soil systems under different nitrogen deposition levels. The short-term effects of N_2_O emissions fluxes and the nitrogen allocation strategies in plant-soil-atmosphere systems are of great theoretical and practical significance for studying global climate change trends and comprehensively and realistically evaluating wetland nitrogen cycling.

## 2. Research materials and test methods

### 2.1 Overview of research sites

The Honghe National Nature Reserve is located in the northeastern part of the Sanjiang Plain in Heilongjiang Province in Northeast China. The geographical position is 47°42′~47°52′ north latitude, 133°34′~133°46′ east longitude, and the total area is 21.84 Mha. The study area has a temperate monsoon climate with a multiyear average temperature of 1.9°C; the coldest month has an average temperature of -23.4°C, and the hottest month has an average temperature of 22.4°C. The extreme minimum temperature is -39.1°C, and the extreme maximum temperature is 40°C. The average annual precipitation is 585 mm, and 50%~70% of the precipitation occurs from July to September. The average annual evaporation is 1166 mm, and the ≥10°C effective accumulated temperature is 2165~2624°C. The number of annual h of sunshine is 2356 hours. The soil types are mainly meadow soil, white soil and marsh soil. The vegetation is Changbai flora, and the zonal vegetation is temperate coniferous and broad-leaved mixed forest. Due to the combined effects of climate, geography, hydrology and other factors, large-area nonzonal swamps, meadows and other low-humidity vegetation areas have formed. The dominant species are grasses and sedges that can tolerate wet, marshy conditions. There are island forests only in some areas. The main types of vegetation are meadows and marshes. The dominant plants include *C*. *angustifolia*, *Glyceria spiculosa*, *Carex lasiocarpa*, and *Carex pseudo-curaica*.

At the experimental station, a typical and representative *C*. *angustifolia* community was selected as the research object. *C*. *angustifolia* is the dominant species, and the coverage is over 80%. The main companion plants are *Sanguisorba parviflora*, *Filipendula intermedia*, *Sium suave*, *Lythrum salicaria*, and *Vicia cracca*, among others.

### 2.2 Experimental design

Simulated nitrogen deposition was achieved by the application of dual-labeled ammonium nitrate (^15^NH_4_^15^NO_3_). Three processing plots (5 m×5 m) were set up at the test station. Each plot was divided into three 1 m×1 m cells, separated by a buffer of a 1 m width. These three cells were randomly set to CK: 0 g nitrogen/m^2^ (control), N1: 4 g nitrogen/m^2^ (low nitrogen), or N2: 8 g nitrogen/m^2^ (high nitrogen).

The N_2_O effluxes were observed using static dark box-gas chromatography. In the early stage of the experiment, the base was buried in advance to allow sufficient time to minimize the disturbance from the base installation. The base was made of stainless steel, length × width × depth = 50 cm× 50 cm × 20 cm, and the upper end of the base had a groove that was 2 cm deep and 2 cm wide for water sealing to prevent air leakage. Holes with a diameter of approximately 2 cm were drilled on the sidewalls of the base in four directions, which facilitated the exchange of soil moisture and nutrients in the base with the soil outside the base. After the base was fixed to the sample, it was not removed until the end of the whole experiment, which helped fix the sampling points and remove the spatial heterogeneity in the sampling.

After the base of the dark box was set up, there were approximately 15 days of stabilization before nitrogen application. After the 15-day stabilization period, ^15^N-labeled ^15^NH_4_^15^NO_3_ was applied.

### 2.3 Measurements of the N_2_O effluxes

The N_2_O effluxes were defined as the change in the measured gas mass discharged in the observation chamber per unit time and area.

$$Formula:\;J=\frac{dc}{dt}\times \frac{M}{V_{0}}\times \frac{P}{P_{0}}\times \frac{T^{0}}{T}\times H$$

*J*——Effluxes (mg · m^-2^ · h^-1^);

$\frac{dcdc}{dtdt}$——The slope of the line in which the concentration of the gas varies with time;

*M*——The molar mass of the gas being measured;

*P*——The pressure at the sampling point;

*T*——Absolute temperature at sampling time;

*V*_*0*_, *P*_*0*_, *T*_*0*_——The molar volume, air pressure and absolute temperature of the gas under standard conditions, respectively;

*H*——Height of sampling tank above water level;

*J*——When is positive, it means release, while negative value means absorption.

^15^N_2_O(mg/m^2^/h) = *J*×(14+15)/45×AT%(^15^N_2_O)×0.01

## 3. Results and analysis

**Fig 1:**
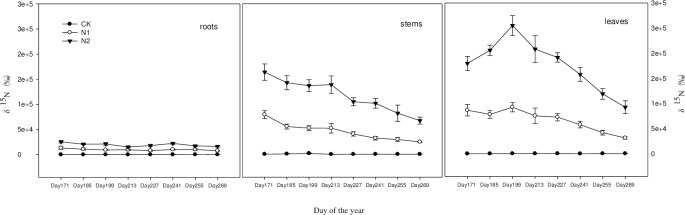
Dynamic changes in nitrogen content in the roots, stems and leaves of *C*. *angustifolia*.

The results of the variance analysis showed that tracer nitrogen was absorbed by plant roots and quickly transferred to plant stems and leaves. The content of tracer nitrogen in the leaves was significantly higher than that in the roots and stems (P < 0.01). It can be seen that most of the tracer nitrogen was absorbed by plant leaves, and the larger the amount of nitrogen applied, the more that was absorbed (Fig 1).

### 3.1 Effect of nitrogen deposition on nitrogen distribution in *C*. *angustifolia*

#### 3.1.1 Nitrogen distribution in the underground part of *C*. *angustifolia*

As shown in Fig 1, the nitrogen content in the roots of *C*. *angustifolia* slowly decreased over the growing season. After analysis of variance, the amount of tracer nitrogen in the roots of the low-nitrogen and high-nitrogen treatments was significantly higher than that in the control (P<0.01), and the amount of tracer nitrogen in the roots of the high-nitrogen treatment (N2) was significantly higher than that of the low-nitrogen treatment (N1) (P< 0.01). Therefore, increasing the amount of nitrogen applied can increase the absorption of tracer nitrogen in the underground part of the plant.

#### 3.1.2 Distribution of nitrogen in the aerial part of *C*. *angustifolia*

As shown in Fig 1, under the low-nitrogen and high-nitrogen treatments, the nitrogen content in the stems and leaves of *C*. *angustifolia* decreased over the growing season. After analysis of variance, the amount of tracer nitrogen in the stem and the leaves was significantly higher than that of the control (P<0.01), and the amount of tracer nitrogen in the stem and the leaves in the high-nitrogen treatment (N2) was significantly higher than that in the low-nitrogen treatment (N1) (P < 0.01). Therefore, increasing the amount of nitrogen applied can greatly increase the absorption of tracer nitrogen in the aerial parts of plants.

### 3.2 Effects of simulated nitrogen deposition on soil nitrogen forms in *C*. *angustifolia* wetlands

**Fig 2:**
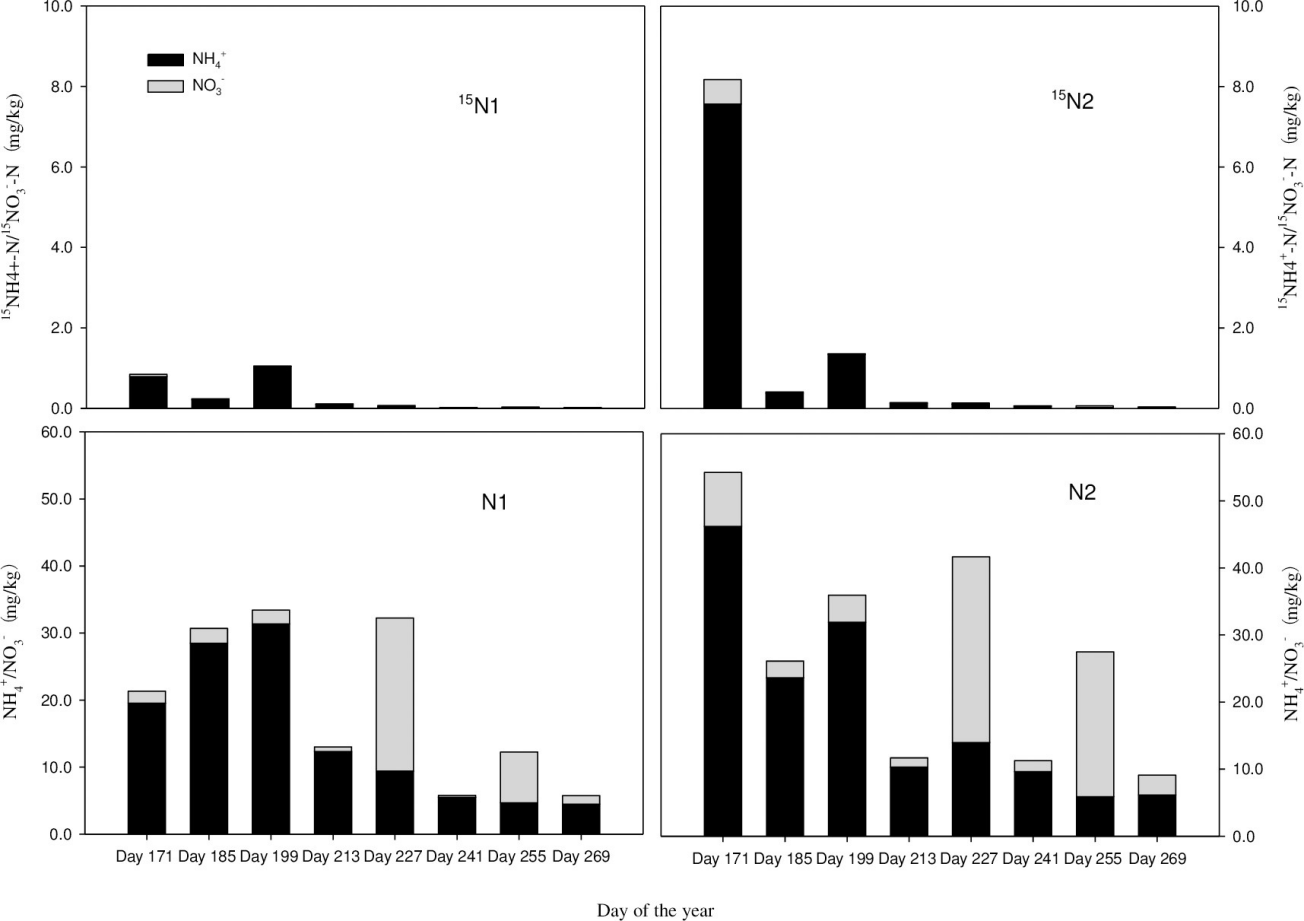
The dynamics of soil nitrogen forms in *C*. *angustifolia* wetlands under different nitrogen treatments.

Fig 2 shows the trends in the NH_4_^+^-N and NO_3_^—^N content in soil under different nitrogen treatments. The nitrogen absorbed by the soil gradually decreased. Except in mid-August and mid-September, relatively more NH_4_^+^-N than NO_3_^—^N was adsorbed by the soil, but the difference was not significant (P>0.05).

Table 1 (^15^N1, ^15^N2) shows that the trace nitrogen in the soil gradually decreased under the different nitrogen treatments, and the amount of ^15^N in ammonium nitrogen adsorbed by the soil in the growing season was higher than the amount of adsorbed ^15^N in nitrate nitrogen. Under the low-nitrogen treatment (Table 1 (^15^N1)), ^15^N in ammonium nitrogen adsorbed by soil reached a maximum value of 1.04 mg/kg in mid-July. Under the high-nitrogen treatment (Table 1 (^15^N2)), ^15^N in ammonium nitrogen adsorbed by soil reached a maximum of 7.56 mg/kg in mid-June and late June, which was more than 7 times the amount adsorbed in the low-nitrogen treatment. In comparison to the low-nitrogen treatment, the high-nitrogen treatment reached a higher maximum adsorption capacity. As the nitrogen application rate increased, the adsorption of nitrogen on the soil increased. Moreover, despite the differences in tracer nitrogen form and addition amount, the soil still adsorbed a large amount of ammonium nitrogen. It was further proven that nitrate nitrogen is mostly absorbed and utilized by plants. Therefore, *C*. *angustifolia* prefers nitrate nitrogen (the same conclusion as that in my articles to be published).

**Table 1:** The content of ^15^N (mg/kg) in ammonium nitrogen and nitrate nitrogen in the soil of the *C*. *angustifolia* wetland under different nitrogen treatments.

	CK (control)		N1 (low nitrogen)		N2 (high nitrogen)	
	NH_4_^+^-^15^N±SE	NO_3_^-^-^15^N±SE	NH_4_^+^-^15^N±SE	NO_3_^-^-^15^N±SE	NH_4_^+^-^15^N±SE	NO_3_^-^-^15^N±SE
**Day 171**	0.18±0.02	0.01±0.00	0.79±0.09	0.05±0.03	7.56±0.89	0.61±0.02
**Day 185**	0.08±0.03	0.02±0.00	0.22±0.04	0.03±0.02	0.38±0.15	0.03±0.02
**Day 199**	0.11±0.04	0.01±0.00	1.04±0.01	0.01±0.00	1.34±0.28	0.02±0.00
**Day 213**	0.05±0.03	0.01±0.00	0.11±0.05	0.01±0.00	0.13±0.09	0.02±0.00
**Day 227**	0.05±0.01	0.02±0.01	0.05±0.01	0.02±0.01	0.10±0.07	0.03±0.01
**Day 241**	0.03±0.02	0.01±0.00	0.02±0.01	0.01±0.00	0.06±0.01	0.02±0.00
**Day 255**	0.01±0.00	0.02±0.01	0.01±0.00	0.02±0.01	0.02±0.01	0.04±0.01
**Day 269**	0.02±0.01	0.01±0.00	0.02±0.01	0.01±0.00	0.03±0.01	0.02±0.00

After applying tracer nitrogen for 15 days, soil adsorption of the tracer nitrogen peaked; that is, under the low-nitrogen treatment, the traced ammonium nitrogen accounted for 4.9% of the total ammonium nitrogen in the soil, and the traced nitrate nitrogen accounted for 3.5% of the total nitrate nitrogen. Under the high-nitrogen treatment, traced ammonium nitrogen accounted for 19.7% of the soil total ammonium nitrogen, and traced nitrate nitrogen accounted for 9.1% of the soil total nitrate nitrogen, further demonstrating the differences between the nitrogen treatments. With more applied nitrogen, the amount of adsorption increased.

### 3.3 Short-term response of ^15^N_2_O emissions flux in the *C*. *angustifolia* community to simulated nitrogen deposition

**Fig 3:**
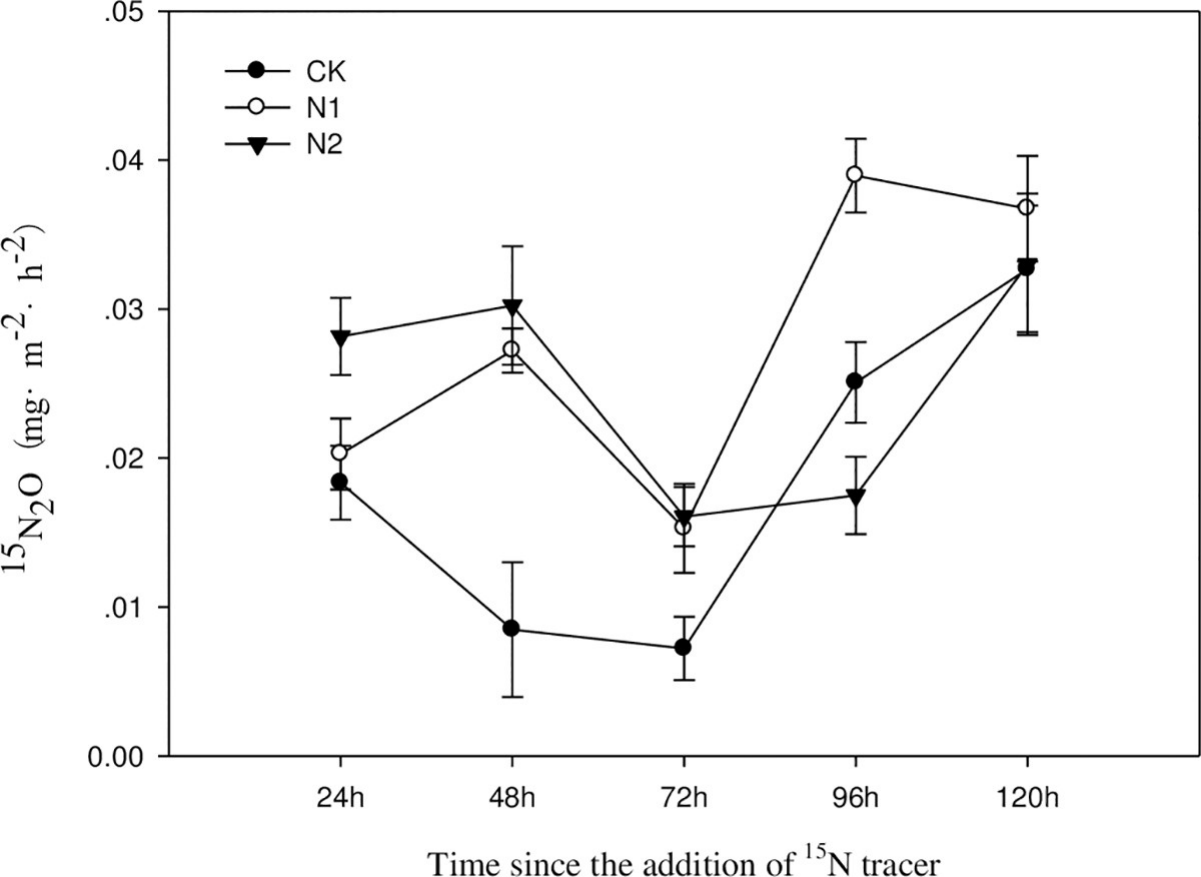
Short-term variation in the ^15^N_2_O emissions flux of the *C*. *angustifolia* community under different nitrogen treatments.

As shown in Fig 3, the variation in the ^15^N_2_O emissions flux under the different nitrogen treatments was consistent. In the short term, ^15^N_2_O showed an “up and down” fluctuation. At 24 h, 48 h and 72 h after the nitrogen application, the high-nitrogen treatment (N2) ^15^N_2_O emissions flux was higher than that of the low-nitrogen treatment flux (N1); at 96 h and 120 h after nitrogen application, the low-nitrogen treatment (N1) ^15^N_2_O emissions flux was higher than that of the high-nitrogen treatment (N2). Except at 96 h, both nitrogen treatments were higher than the control (CK). Therefore, nitrogen application increased the ^15^N_2_O emissions flux, but the difference between the low-nitrogen and high-nitrogen treatments was not significant (P>0.05). The maximum value of the ^15^N_2_O emissions flux was 0.09% of the total N_2_O emissions flux, indicating that most of the exogenous nitrogen was absorbed and utilized by the soil.

**Fig 4:**
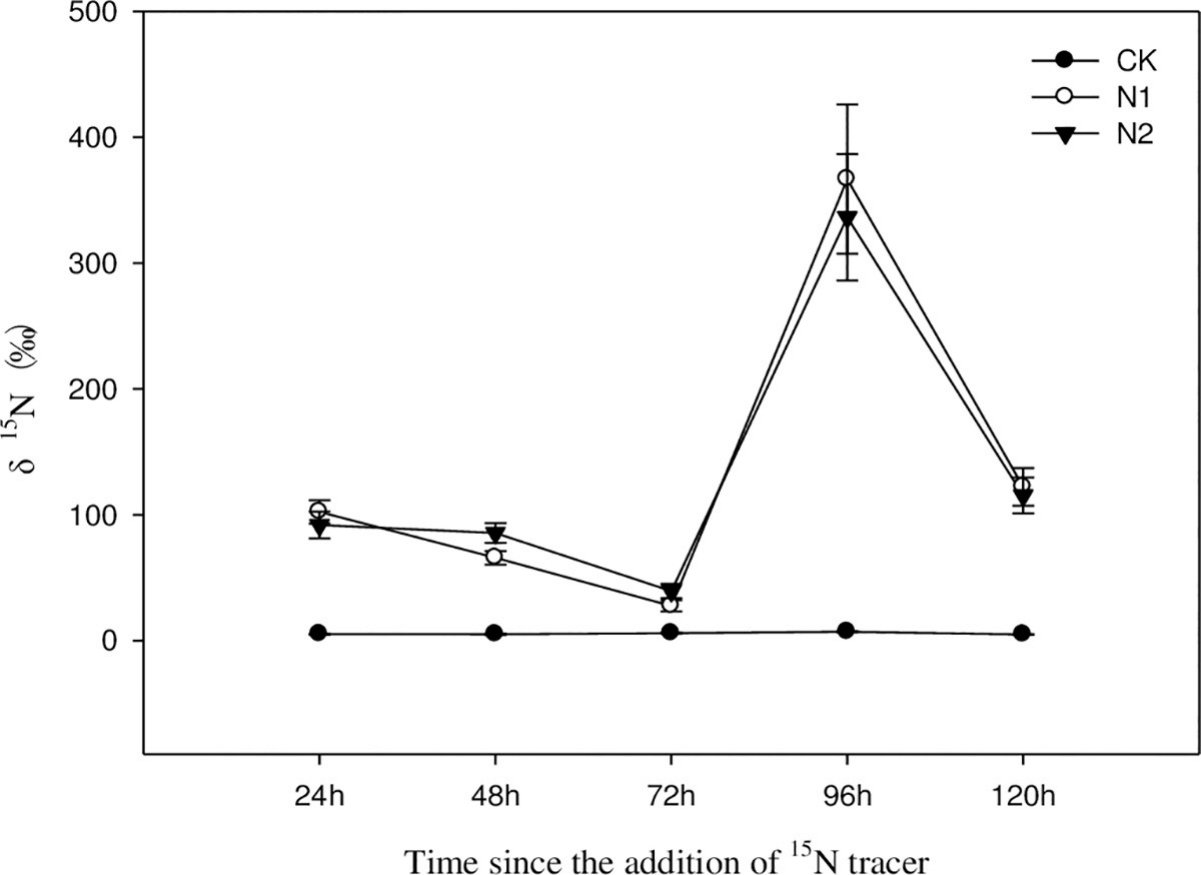
Short-term variation in tracer nitrogen δ value in N_2_O of the *C*. *angustifolia* community under different nitrogen treatments.

As shown in Fig 4, the variation in the tracer nitrogen δ value in N_2_O emitted under different nitrogen treatments was almost the same among treatments. The differences at different times and in different treatments were not significant (P>0.05), but the values for the low-nitrogen and high-nitrogen treatments were higher than that for the control; in addition, the value for the high-nitrogen treatment was significantly higher than that for the control (P<0.05). Therefore, as the amount of nitrogen applied increased, the tracer nitrogen in the discharged N_2_O also increased, further demonstrating that nitrogen application can increase N_2_O emissions flux.

## 4. Discussion

Nitrogen is an important limiting factor for the growth of terrestrial plants. Nitrogen input can directly damage plant tissues [14] or affect the nutrient absorption and distribution of plants by changing the physical and chemical properties of the soil, resulting in changes in the biomass allocation characteristics of various organs of plants [15,16,17]. In this study, the isotope tracing technique was used and showed that different nitrogen deposition levels have a certain promotion effect on the aboveground and underground parts of *C*. *angustifolia*, which is consistent with the results of Liu Deyan et al. (2008) [18]. Because nitrogen is an important limiting factor for plant growth in the Sanjiang Plain wetland [19], an appropriate amount of nitrogen input can stimulate plant growth. Domestic scholars have also studied the response characteristics of nitrogen input in the estuary wetland of the Minjiang River. It was also found that nitrogen input promoted the TN content of roots, stems, leaves and sheaths of *S*. *alterniflora* [17]. The study also found that most of the tracer nitrogen was absorbed and utilized by the plant body, and the absorption amount accounted for 52%-86% of the total tracer amount applied. The absorption amount decreased linearly with the growing season. The higher the nitrogen application was, the more the plant body absorbed. With less volatilization and leaching loss, increased nitrogen application can greatly improve nitrogen utilization.

The form of nitrogen greatly affected the absorption characteristics of nitrate nitrogen and ammonium nitrogen in the plant roots. Under the same nitrogen application rate, the amount of nitrate nitrogen absorbed by the plant roots was much greater than the amount of ammonium nitrogen absorbed, and most of the ammonium nitrogen was adsorbed by the soil. The more nitrogen applied, the more ammonium nitrogen is adsorbed by the soil. However, the amount of traced nitrate nitrogen adsorbed by the soil was very small, generally accounting for 1%-2% of the total tracer nitrogen, and most of the adsorption was traced ammonium. The study found that the amount of tracer nitrogen in the soil did not change significantly with the growing season.

N_2_O emissions from wetland ecosystems are mainly determined by nitrification and denitrification processes in the soil. These processes are strongly influenced by soil temperature and humidity, NH_4_^+^-N, NO_3_^—^N in the soil, and soil microbes [20,21,22,23]. Previous studies have shown that the effects of nitrogen application on N_2_O emissions from wetland ecosystems can vary. Hu et al. [24] showed that in the short term, nitrogen deposition did not change the seasonal variation and diurnal variation in the soil N_2_O flux of a deciduous broad-leaved forest in the northern subtropical zone. Jiang et al. [25] studied alpine meadows on the Qinghai-Tibet Plateau and found that the short-term addition of nitrogen in the growing season led to an increase in N_2_O emissions. The reason may be that nitrogen application promoted the denitrification process in the alpine meadows and promoted N_2_O emissions. In addition, Fang et al. [26] found that low nitrogen did not change soil N_2_O emissions, while high nitrogen promoted N_2_O emissions.

This outcome was due to the changes in the community structure of N_2_O-producing bacteria under high-nitrogen conditions, which result in an increase in their activity and thereby promote N_2_O emissions. The study found that wetland swampy meadows are a weak N_2_O emissions source, and different nitrogen treatments can significantly promote N_2_O emissions in wetlands within 72 h. After 96 h, the emissions promotion from low nitrogen disappears, but high nitrogen levels still promote N_2_O emissions. This may be because the input nitrogen is mainly absorbed and utilized by other plants, and the amount of nitrogen remaining in the soil is insufficient to change the rate of soil nitrification and denitrification. Therefore, the effect of low-nitrogen input on soil N_2_O emissions is not obvious. This conclusion is related to that of Mou Xiaojie et al. [27]. The conclusions from the study of the Minjiang River estuary wetland are consistent, depending on the nitrification and denitrification processes, and may be affected by time, soil temperature and humidity, and soil nutrient content [28].

## Supporting information

S1 Data (XLSX)
